# Towards a modular and scalable holographic display

**DOI:** 10.1038/s41377-022-00786-9

**Published:** 2022-04-19

**Authors:** Pierre-Alexandre Blanche

**Affiliations:** Wyant College of Optical Sciences, University of Arizona, Tucson, AZ USA

**Keywords:** Imaging and sensing, Optics and photonics

## Abstract

Holographic three-dimensional (3D) display can be made very large using a modular system that allows seamless spatial tiling of multiple coarse integral holographic images.

The principle of holography is known since 1948 with the publication of Dennis Gabor^1,2^ works on the correction of aberration observed in electron microscopy. The first recording of holographic images happens in 1961 and is credited to both the group of Leith and Upatnieks^3^ as well as Denisyuk^4^. This optical recording of holograms was enabled by the invention of the laser a year earlier^5^. Since then, researchers have looked at the way to reproduce holographic images using a dynamic system so a holographic television can be created. This is not a simple academic exercise, but a matter of achieving the ultimate 3D display^6,7^. Indeed, holography is a technique that can reproduce all the necessary visual cues to truly present 3D image^8,9^. These cues include motion parallax, occlusions, and accommodation^10^.

Even though immense progresses have been made in the computation of holograms^11,12^, the optical reproduction of these 3D holograms has still been lacking. The main reason for this difficulty is the absence of an electronic display that satisfies all the conditions needed for the high fidelity rendering of holograms. Such a display should have an extremely high number of pixels (about 2 × 10^12^), and a pixel pitch on the order of the wavelength (0.5 µm)^8^. A lesser pixel count will reduce the resolution of the holographic image, and a larger pixel pitch will reduce the field of view of the observed image. To give a sense of scale, a 4 K UHD display has 8 × 10^6^ pixels, and the smallest pixel pitch in microdisplay is around a few micrometers.

In a recent paper published by Jin Li, Quinn Smithwick, and Daping Chu^13^, the authors are taking the approach of making a modular holographic display that can eventually be scaled up by adding more and more elements. Dubbed the “Holobricks,” a single element of the display has a limited filed of view by itself, but it can be extended by stacking additional elements side by side (Figure 1 of ref. ^13^). At the heart of this display is the Texas Instruments DMD, which has an image refresh rate higher than commercially available liquid crystal micropanels. This high refresh rate is taken advantage of by the authors to reproduce three colors (red, green, and blue) using temporal multiplexing.One of the difficulties that the authors had to overcome was to make sure that there was no edge artifact where the hologram merges. A combination of pre-compensation calibration method for the hardware and correcting the reminding error during the computation of the hologram was used to obtain a good display quality (Figure 5 of ref. ^13^).

Although the technique could be set to reproduce accommodation as well as parallax using layer-based computer generated hologram, the first demonstrator presented in the manuscript only demonstrates an occlusion capable multiview hologram, which does not include accommodation cues.

Compared to previous systems that use a modular approach, such as the active tiling holographic display introduced by Slinger et al.^14^, each holobrick is only using a single microdisplay, bypassing the dual stage addressing (first electronically addressed SLM, then optically addressed SLM) used in the past. This make the Holobricks more straightforward to assemble, and potentially more affordable to build.

Some more works is still needed to improve the image quality displayed by the Holobricks, such as eliminating the speckle noise still present in the hologram. However, the authors hope that this work could pave the way to an ultra-large-size and large-viewing-angle holographic displays. Based on the specific application requirements, the Holobricks can be designed into different holographic display configuration, such as holographic video displays, holographic video walls, or holographic interactive kiosks, etc.
